# Supplementary material for: Antimicrobial resistance and AmpC production in ESBL-producing Klebsiella pneumoniae and Klebsiella quasipneumoniae: A retrospective study in Japanese clinical isolates
Source: PLoS One. 2024 May 14;19(5):e0303353. doi: 10.1371/journal.pone.0303353 (PMC11093370; doi:10.1371/journal.pone.0303353)
Supplement: Attachment — Submitted filename: PONE-D-24-04229 REVIEW.pdf [file pone.0303353.s004.pdf]

PLOS ONE

Antimicrobial resistance and AmpC production in ESBL-producing Klebsiella pneumoniae and K. quasipneumoniae subsp. similipneumoniae: A retrospective study in Japanese clinical isolates

--Manuscript Draft--

**Manuscript Number:**	PONE-D-24-04229
**Article Type:**	Research Article
**Full Title:**	Antimicrobial resistance and AmpC production in ESBL-producing Klebsiella pneumoniae and K. quasipneumoniae subsp. similipneumoniae: A retrospective study in Japanese clinical isolates
**Short Title:**	Antimicrobial resistance and AmpC production in ESBL-producing K. pneumoniae and K. quasipneumoniae
**Corresponding Author:**	Naoki Watanabe<br>Kameda Medical Center<br>Kamogawa, Chiba JAPAN
**Keywords:**	AmpC; antimicrobial resistance; extended spectrum beta lactamase; Klebsiella pneumoniae; Klebsiella quasipneumoniae; Klebsiella variicola
**Abstract:**	The aim of this study is to evaluate the prevalence of various species, ESBL genotypes, antimicrobial resistance patterns, and AmpC co-production in ESBL-producing Klebsiella.<br>In total, 139 ESBL-producing Klebsiella clinical isolates were collected in Japan, along with their antimicrobial susceptibility profiles. Isolates were identified using a web-based tool. The ESBL genes in isolates were detected via multiplex PCR. Screening for AmpC-producing strains was conducted using cefoxitin disks, followed by multiplex PCR for AmpC gene detection. We analyzed the antimicrobial resistance patterns across major ESBL genotypes.<br>The web-based tool identified 135 isolates (97.1%) as Klebsiella pneumoniae and 4 (2.9%) as Klebsiella quasipneumoniae subsp. similipneumoniae, with no Klebsiella variicola detected. The CTX-M-1 group was the predominant genotype in K. pneumoniae (83/135, 61.5%) and K. quasipneumoniae subsp. similipneumoniae (3/4, 75.0%). The CTX-M-9 group was the second most prevalent in K. pneumoniae (45/135, 33.3%). In K. pneumoniae, all isolates showed susceptibility to imipenem, meropenem, and amikacin. High resistance rates to quinolones (46.7-63.0%) and trimethoprim/sulfamethoxazole (78.5%) were observed. The CTX-M-1 group exhibited higher resistance to ciprofloxacin (66/83, 79.5%) compared to the CTX-M-9 group (18/45, 40.0%). A similar trend was observed for levofloxacin and trimethoprim/sulfamethoxazole. Among the sixteen isolates showing AmpC screening positive, only one K. pneumoniae isolate (0.7%) was confirmed to carry the AmpC gene.<br>K. pneumoniae harboring the CTX-M1 group was predominant in Japanese ESBL-producing isolates, with AmpC-producing isolates being rare. These isolates demonstrated significant resistance to quinolones and trimethoprim/sulfamethoxazole, particularly in the CTX-M1 group.
**Order of Authors:**	Naoki Watanabe
	Tomohisa Watari
	Yoshihito Otsuka
	Masahiko Ito
	Kazufumi Yamagata
	Miyuki Fujioka
**Additional Information:**	
**Question**	**Response**
**Financial Disclosure**	The author(s) received no specific funding for this work.

Powered by Editorial Manager® and ProduXion Manager® from Aries Systems Corporation

Enter a financial disclosure statement that describes the sources of funding for the work included in this submission. Review the submission guidelines for detailed requirements. View published research articles from *PLOS ONE* for specific examples.<br><br>This statement is required for submission and **will appear in the published article** if the submission is accepted. Please make sure it is accurate.<br><br>**Funded studies**<br>Enter a statement with the following details:<br>• Initials of the authors who received each award<br>• Grant numbers awarded to each author<br>• The full name of each funder<br>• URL of each funder website<br>• Did the sponsors or funders play any role in the study design, data collection and analysis, decision to publish, or preparation of the manuscript?<br><br>Did you receive funding for this work?	
**Competing Interests**<br><br>Use the instructions below to enter a competing interest statement for this submission. On behalf of all authors, disclose any competing interests that could be perceived to bias this work—acknowledging all financial support and any other relevant financial or non-financial competing interests.<br><br>This statement is required for submission and **will appear in the published article** if the submission is accepted. Please make sure it is accurate and that any funding sources listed in your Funding Information later in the submission form are also declared in your Financial Disclosure statement.	The authors have declared that no competing interests exist.

Powered by Editorial Manager® and ProduXion Manager® from Aries Systems Corporation

View published research articles from *PLOS ONE* for specific examples.<br><br>**NO authors have competing interests**<br><br>Enter: *The authors have declared that no competing interests exist.*<br><br>**Authors with competing interests**<br><br>Enter competing interest details beginning with this statement:<br><br>*I have read the journal's policy and the authors of this manuscript have the following competing interests: [insert competing interests here]*<br><br>* typeset	
**Ethics Statement**<br><br>Enter an ethics statement for this submission. This statement is required if the study involved:<br><br>• Human participants<br>• Human specimens or tissue<br>• Vertebrate animals or cephalopods<br>• Vertebrate embryos or tissues<br>• Field research<br><br>Write "N/A" if the submission does not require an ethics statement.<br><br>General guidance is provided below. Consult the submission guidelines for detailed instructions. **Make sure that all information entered here is included in the Methods section of the manuscript.**	This study involved only isolates and their associated data, without including any patient information linked to the isolates. Consequently, the Research Ethics Committee of the Kameda Medical Center in Japan, determined that ethical review and informed consent were not required for this research.

**Format for specific study types**<br><br>**Human Subject Research (involving human participants and/or tissue)**<br>• Give the name of the institutional review board or ethics committee that approved the study<br>• Include the approval number and/or a statement indicating approval of this research<br>• Indicate the form of consent obtained (written/oral) or the reason that consent was not obtained (e.g. the data were analyzed anonymously)<br><br>**Animal Research (involving vertebrate animals, embryos or tissues)**<br>• Provide the name of the Institutional Animal Care and Use Committee (IACUC) or other relevant ethics board that reviewed the study protocol, and indicate whether they approved this research or granted a formal waiver of ethical approval<br>• Include an approval number if one was obtained<br>• If the study involved *non-human primates*, add *additional details* about animal welfare and steps taken to ameliorate suffering<br>• If anesthesia, euthanasia, or any kind of animal sacrifice is part of the study, include briefly which substances and/or methods were applied<br><br>**Field Research**<br><br>Include the following details if this study involves the collection of plant, animal, or other materials from a natural setting:<br>• Field permit number<br>• Name of the institution or relevant body that granted permission	
**Data Availability**<br><br>Authors are required to make all data underlying the findings described fully available, without restriction, and from the time of publication. PLOS allows rare exceptions to address legal and ethical concerns. See the PLOS Data Policy and FAQ for detailed information.	Yes - all data are fully available without restriction

A Data Availability Statement describing where the data can be found is required at submission. Your answers to this question constitute the Data Availability Statement and **will be published in the article**, if accepted.<br><br>**Important:** Stating 'data available on request from the author' is not sufficient. If your data are only available upon request, select 'No' for the first question and explain your exceptional situation in the text box.<br><br>Do the authors confirm that all data underlying the findings described in their manuscript are fully available without restriction?	
**Describe where the data may be found in full sentences. If you are copying our sample text, replace any instances of XXX with the appropriate details.**<br><br>• If the data are **held or will be held in a public repository**, include URLs, accession numbers or DOIs. If this information will only be available after acceptance, indicate this by ticking the box below. For example: *All XXX files are available from the XXX database (accession number(s) XXX, XXX.).*<br>• If the data are all contained **within the manuscript and/or Supporting Information files**, enter the following: *All relevant data are within the manuscript and its Supporting Information files.*<br>• If neither of these applies but you are able to provide **details of access elsewhere**, with or without limitations, please do so. For example:<br><br>*Data cannot be shared publicly because of [XXX]. Data are available from the XXX Institutional Data Access / Ethics Committee (contact via XXX) for researchers who meet the criteria for access to confidential data.*<br><br>*The data underlying the results presented in the study are available from (include the name of the third party*	All relevant data are within the manuscript and its Supporting Information files.

and contact information or URL).<br>• This text is appropriate if the data are owned by a third party and authors do not have permission to share the data.<br><br>* typeset	
Additional data availability information:	Tick here if the URLs/accession numbers/DOIs will be available only after acceptance of the manuscript for publication so that we can ensure their inclusion before publication.

Powered by Editorial Manager® and ProduXion Manager® from Aries Systems Corporation

Manuscript Click here to access/download;Manuscript;Manuscript.docx

Antimicrobial resistance and AmpC production in ESBL-producing *Klebsiella pneumoniae* and *K. quasipneumoniae* subsp. *similipneumoniae*: A retrospective study in Japanese clinical isolates

Naoki Watanabe [1, 2, *], Tomohisa Watari [1], Yoshihito Otsuka [1], Masahiko Ito [3], Kazufumi Yamagata [2], Miyuki Fujioka [2],

[1] Department of Clinical Laboratory, Kameda Medical Center, Higashi-cho 929, Kamogawa, Chiba, Japan

[2] Graduate School of Health Sciences, Hirosaki University, Hon-cho 66-1, Hirosaki, Aomori, Japan

[3] Sapporo Clinical Laboratory Inc., 2-2, Kita 3 Nishi 18, Chuo-ku, Sapporo, Hokkaido, Japan

*** Corresponding author**

E-mail: watanabe.naoki.4@kameda.jp (NW)

Short title: Antimicrobial resistance and AmpC production in ESBL-producing *K. pneumoniae* and *K. quasipneumoniae*

Abstract

The aim of this study is to evaluate the prevalence of various species, ESBL genotypes, antimicrobial resistance patterns, and AmpC co-production in ESBL-producing *Klebsiella*.

In total, 139 ESBL-producing *Klebsiella* clinical isolates were collected in Japan, along with their antimicrobial susceptibility profiles. Isolates were identified using a web-based tool. The ESBL genes in isolates were detected via multiplex PCR. Screening for AmpC-producing strains was conducted using cefoxitin disks, followed by multiplex PCR for AmpC gene detection. We analyzed the antimicrobial resistance patterns across major ESBL genotypes.

The web-based tool identified 135 isolates (97.1%) as *Klebsiella pneumoniae* and 4 (2.9%) as *Klebsiella quasipneumoniae* subsp. *similipneumoniae*, with no *Klebsiella variicola* detected. The CTX-M-1 group was the predominant genotype in *K. pneumoniae* (83/135, 61.5%) and *K. quasipneumoniae* subsp. *similipneumoniae* (3/4, 75.0%). The CTX-M-9 group was the second most prevalent in *K. pneumoniae* (45/135, 33.3%). In *K. pneumoniae*, all isolates showed susceptibility to imipenem, meropenem, and amikacin. High resistance rates to quinolones (46.7-63.0%) and trimethoprim/sulfamethoxazole (78.5%) were observed. The CTX-M-1 group exhibited higher resistance to ciprofloxacin (66/83, 79.5%) compared to the CTX-M-9 group (18/45, 40.0%). A similar trend was observed for levofloxacin and trimethoprim/sulfamethoxazole. Among the sixteen isolates showing AmpC screening positive, only one *K. pneumoniae* isolate (0.7%) was confirmed to carry the AmpC gene.

K. pneumoniae harboring the CTX-M1 group was predominant in Japanese ESBL-producing isolates, with AmpC-producing isolates being rare. These isolates demonstrated significant resistance to quinolones and trimethoprim/sulfamethoxazole, particularly in the CTX-M1

group.

Introduction

Klebsiella pneumoniae is a Gram-negative bacillus belonging to the Enterobacteriaceae family [1]. Recently, multidrug-resistant *K. pneumoniae* has emerged as a global concern. *K. pneumoniae* isolates that are resistant to extended-spectrum β-lactamases (ESBL) and carbapenems have been recognized as significant public health threats. Various clones, including clonal groups 258, 14 [2], ST307, and 147 [3, 4] are associated with multidrug-resistant *K. pneumoniae*.

In ESBL-producing *K. pneumoniae*, the CTX-M1 group, particularly CTX-M-15, is the most common ESBL genotype. CTX-M-15 is prevalent in various regions including the United States [5], Canada [6], Europe [7, 8], Asia [9], and Africa [10]. Moreover, ESBL-producing isolates can co-produce AmpC. A study conducted in Iran reported a high prevalence of co-producing AmpC isolates in ESBL-producing *K. pneumoniae* [11]. However, research on ESBL and AmpC co-producing *K. pneumoniae* is limited.

K. pneumoniae has closely related species such as *Klebsiella quasipneumoniae* and *Klebsiella variicola*, which include subspecies like *K. quasipneumoniae* subsp. quasipneumoniae and *K. quasipneumoniae* subsp. *similipneumoniae* [12]. ESBL production has also been observed in *K. quasipneumoniae* and *K. variicola* [13, 14]. However, owing to the frequent misidentification of *K. quasipneumoniae* and *K. variicola* as *K. pneumoniae* in clinical settings [15], the global epidemiology of ESBL-producing *K. quasipneumoniae* and *K. variicola* is ill-understood.

This study aims to determine the prevalence of various *Klebsiella* species, ESBL genotypes, antimicrobial resistance, and AmpC co-production in clinical isolates of ESBL-producing *K. pneumoniae*. Additionally, we compared antimicrobial resistance among different

Klebsiella species and among major ESBL genotypes. The findings are expected to provide updated insights into ESBL-producing *Klebsiella* and their antimicrobial resistance patterns. These insights will be invaluable for clinicians and researchers in understanding the dynamics of ESBL-producing *K. pneumoniae*, as well as the characteristics of *K. quasipneumoniae* and *K. variicola*.

Material and methods

We conducted a retrospective study on ESBL-producing *Klebsiella* in Japan. In this study, we collected clinical isolates of ESBL-producing *Klebsiella* isolates and investigated the following factors: the frequency of each *Klebsiella* species, the distribution of ESBL genotypes, the comparison of antimicrobial resistance between different *Klebsiella* species as well as between the major genotypes, and the presence of AmpC beta-lactamase. This study involved only isolates and their associated data, without including any patient information linked to the isolates. Consequently, the Research Ethics Committee of the Kameda Medical Center in Japan, determined that ethical review and informed consent were not required for this research.

Isolate collection

In total, we collected 139 ESBL-producing *Klebsiella* isolates from two locations in Japan: Kameda Medical Center in the eastern part and Sapporo Clinical Laboratory in the northern part. At Kameda Medical Center, ninety isolates were obtained between February 2020 and November 2023, while forty-nine isolates were collected at Sapporo Clinical Laboratory from

October to November 2023. In cases where multiple ESBL-producing *K. pneumoniae* isolates were obtained from the same patient, only the initial isolate was included in the study. These isolates were identified as either *K. pneumoniae* or *K. variicola* using matrix-assisted laser desorption ionization-time of flight mass spectrometry (MALDI Biotyper, Bruker Daltonik GmbH, Germany). The ESBL phenotype was confirmed using the Double-disk synergy test [16,17]. Isolates were stored at -80°C until further identification of *Klebsiella* species, detection of ESBL genotypes, AmpC screening, and carbapenemase screening. For each test, isolates were cultured on Trypticase Soy Agar with 5% Sheep Blood (Becton Dickinson, USA) under aerobic conditions.

Identification of *Klebsiella* strains

Routine testing often does not distinguish between closely related *Klebsiella* species such as *K. quasipneumoniae* and *K. variicola*. To address this, we used the *Klebsiella* MALDI TypeR [18], a web-based tool for identifying *Klebsiella*. This tool involves obtaining the mass spectra of isolates and analyzing them through a web-based platform. A single colony was selected using a toothpick and applied to a target plate. Then, one µL of matrix solution (α-cyano-4-hydroxycinnamic acid [Bruker Daltonik GmbH], dissolved in acetonitrile/water/trifluoroacetic acid [50:47.5:2.5 v/v, Sigma-Aldrich, USA]), was added to the colony smear and allowed to air-dry. Mass spectra were acquired using a microflex LT/SH and flexControl Version 3.4 (Bruker Daltonik GmbH). Species identification was conducted by uploading the mass spectra

to https://maldityper.pasteur.fr/ for analysis.

Antimicrobial susceptibility testing

Antimicrobial susceptibility testing was conducted using the broth dilution method with an EIKEN dry plate (Eiken Chemical, Japan). The test encompassed fourteen antibiotics, including amoxicillin-clavulanate, piperacillin-tazobactam, ceftazidime, ceftriaxone, cefepime, cefmetazole, imipenem, meropenem, gentamicin, tobramycin, amikacin, ciprofloxacin, levofloxacin, and trimethoprim/sulfamethoxazole (TMP-SMX). The assay conditions and interpretation of results were adherent to the Clinical and Laboratory Standards Institute M100 [19]. Quality control was assured using the E. coli ATCC 25922 isolate. Data on Antimicrobial susceptibility was collected and validated after confirming the absence of any discrepancies in the quality control process.

Screening test for AmpC and carbapenemase production

The screening test for AmpC was conducted using the cefoxitin disk susceptibility test as outlined by the European Committee on Antimicrobial Susceptibility Testing [20]. BD BBL Sensi-Disc Cefoxitin FOX-30 (Becton Dickinson, USA) was utilized, and a zone diameter of less than 19 mm around the cefoxitin disc indicated a positive result for AmpC screening. The screening test for carbapenemase was performed using the modified Carbapenem Inactivation Method against isolates with a minimum inhibitory concentration (MIC) of ≥ 2 for either imipenem or meropenem, according to the Clinical and Laboratory Standards Institute M100 [19]. The m-CIM procedure and interpretation were adherent to the Clinical and Laboratory

Standards Institute M100 [19].

Detection of AmpC and carbapenemase genotypes

Template DNA was prepared using the Cica GeneusR DNA Extraction Reagent (Kanto Chemical, Japan). The ESBL and AmpC genotypes were confirmed using the Cica GeneusR ESBL Genotype Detection Kit 2 and Cica GeneusR AmpC Genotype Detection Kit 2, respectively (both from Kanto Chemical). These kits were utilized according to the manufacturer's instructions. The Cica GeneusR ESBL Genotype Detection Kit 2 is a multiplex PCR kit that targets ESBL genes, including the CTX-M group (M-1, M-2, M-8, M-9, M-25, and M chimera), SHV, GES, and TEM. The Cica GeneusR AmpC Genotype Detection Kit 2 targets the AmpC genes, including the CIT, FOX, ACT, DHA, ACC, and MOX families. Multiplex PCR was performed using the GeneAtlas TypeG thermal cycler (Astec, Japan). Primers provided with the kits were used, and the PCR protocol involved 30 cycles at 94 °C for 15 s, 63 °C for 15 s, and 72 °C for 40 s. PCR products were electrophoresed on a 2.0% agarose gel in 1 × Tris-acetate-EDTA buffer and stained with 1.0% ethidium bromide.

Statistical analysis

Statistical analysis was conducted using EZR version 1.54 [21], with statistical significance set at $P < 0.05$. We calculated the percentage and 95% confidence interval (95% CI) for each of the following variables: the prevalence of each *Klebsiella* species within the ESBL-producing *Klebsiella*, the proportion of AmpC-producing isolates within the ESBL-producing *Klebsiella*, and the frequency of carbapenemase-producing isolates within the ESBL-producing *Klebsiella*.

Furthermore, comparisons of categorical variable ratios between two groups were performed using Fisher's exact test. This included analyzing the resistance ratio to each antibiotic for each *Klebsiella* species, the ratio of ESBL genotypes among each *Klebsiella* species, and the resistance ratio to each antibiotic among the major genotypes.

Results

Prevalence of *K. pneumoniae*, *K. quasipneumoniae*, and *K. variicola* among the ESBL-producing *Klebsiella* isolates

The isolation specimens and characteristics of the ESBL-producing *Klebsiella* isolates are presented in Table 1. Most of the ESBL-*Klebsiella* isolates were obtained from urine specimens (74/139, representing 53.2%), followed by sputum (35/139, 25.2%), and blood (15/139, 10.8%). Using *Klebsiella* MALDI TypeR for identification, 135 isolates (97.1%, [95% CI, 92.8-99.2%]) were classified as *K. pneumoniae* and four (2.9%, [95% CI, 0.8-7.2%]) as *K. quasipneumoniae* subsp. *similipneumoniae*. No *K. variicola* was detected in the ESBL-producing *Klebsiella* isolates (95% CI, 0.0-2.6%). Comprehensive data on the isolates and *Klebsiella* MALDI TypeR results are presented in Table S1.

Approximate locations for Table 1.

Table 1. Characteristics of ESBL-producing *K. pneumoniae* and *K. quasipneumoniae* subsp. *similipneumoniae*

**Characteristics**	**Group of *Klebsiella* species**			**P value**
	**All (*n* = 139)**	***K. pneumoniae* (*n* = 135)**	***K. quasipneumoniae* (*n* = 4)**	
Specimen, no. (%)				0.7
Urine	74 (53.2)	72 (53.3)	2 (50.0)	
Sputum	35 (25.2)	35 (25.2)	1 (25.0)	
Blood	15 (10.8)	14 (10.4)	1 (25.0)	
Wound	5 (3.6)	5 (3.7)	0 (0.0)	
Stool	3 (2.2)	3 (2.2)	0 (0.0)	
Other*	7 (5.0)	7 (5.2)	0 (0.0)	
Number of ESBL-gene, no. (%)				< 0.001
One	7 (5.0)	4 (3.0)	3 (75.0)	< 0.001
Two	48 (34.5)	47 (34.8)	1 (25.0)	1
Three	84 (60.4)	84 (62.2)	0 (0.0)	0.02
Screening test, positive no. (%)				
AmpC	17 (12.2)	16 (11.9)	1 (25.0)	0.4
Carbapenemase	0 (0.0)	0 (0.0)	0 (0.0)	NA

Description: Table 1 presents the characteristics of 139 ESBL-producing *Klebsiella* isolates. Using *Klebsiella* MALDI TypeR, 135 isolates were identified as *K. pneumoniae* and four as *K. quasipneumoniae* subsp. *similipneumoniae*, with no *K. variicola* detected.

Footnotes: *Other specimens included two ascites, two bile, two catheters, and one peritoneal dialysis fluid.

K. quasipneumoniae, *K. quasipneumoniae* subsp. *similipneumoniae*; NA, not applicable.

Antimicrobial susceptibilities of *K. pneumoniae* and *K. quasipneumoniae* subsp. *similipneumoniae*

The antimicrobial susceptibilities of *K. pneumoniae* and *K. quasipneumoniae* subsp. *similipneumoniae* are detailed in Table 2. *K. pneumoniae* showed resistance to amoxicillin-clavulanate in 5.2% of cases (7/135) and to piperacillin-tazobactam in 18.5% (25/135) of them. In contrast, *K. quasipneumoniae* subsp. *similipneumoniae* did not exhibit resistance to these antibiotics. Neither *K. pneumoniae* nor *K. quasipneumoniae* subsp. *similipneumoniae* isolates were resistant to imipenem or meropenem. Resistance to cefmetazole was found in 3% of *K. pneumoniae* isolates (4/135) but was absent in *K. quasipneumoniae* subsp. *similipneumoniae* (0/4).

High resistance to fluoroquinolones and TMP-SMX was observed in both *K. pneumoniae* and *K. quasipneumoniae* subsp. *similipneumoniae*. The resistance rates for levofloxacin were 46.7% in *K. pneumoniae* (63/135) and 25% in *K. quasipneumoniae* subsp. *similipneumoniae* (1/4), while for ciprofloxacin, they were 63.0% in *K. pneumoniae* (85/135) and 25% in *K. quasipneumoniae* subsp. *similipneumoniae* (1/4). The resistance rates to TMP-SMX were 78.5% (106/135) in *K. pneumoniae* and 50.0% (2/4) in *K. quasipneumoniae* subsp. *similipneumoniae*. No significant differences in antimicrobial susceptibilities were noted between *K. pneumoniae* and *K. quasipneumoniae* subsp. *similipneumoniae*.

Approximate locations for Table 2.

Table 2. Antimicrobial resistant isolates of ESBL-producing *K. pneumoniae* and *K. quasipneumoniae* subsp. *similipneumoniae*

**Antibiotic**	**Resistant isolates, no. (%)**		**P value**
	***K. pneumoniae* (*n* = 135)**	***K. quasipneumoniae* (*n* = 4)**	
β-Lactam combination			
Amoxicillin-clavulanate	7 (5.2)	0 (0.0)	1
Piperacillin-tazobactam	25 (18.5)	0 (0.0)	1
Cephem			
Ceftazidime	68 (50.4)	3 (75.0)	0.6
Ceftriaxone	131 (97.0)	4 (100)	1
Cefepime	96 (71.1)	3 (75.0)	1
Cefmetazole	4 (3.0)	0 (0.0)	1
Carbapenem			
Imipenem	0 (0.0)	0 (0.0)	NA
Meropenem	0 (0.0)	0 (0.0)	NA
Aminoglycoside			
Gentamicin	48 (35.6)	0 (0.0)	0.3
Tobramycin	23 (17.0)	0 (0.0)	1

Amikacin	0 (0.0)	0 (0.0)	NA
Fluoroquinolone			
Levofloxacin	63 (46.7)	1 (25.0)	0.6
Ciprofloxacin	85 (63.0)	1 (25.0)	0.2
Folate pathway			
TMP-SMX	106 (78.5)	2 (50.0)	0.2

Description: Table 2 provides the antimicrobial susceptibility profiles of *K. pneumoniae* and *K. quasipneumoniae* subsp. *similipneumoniae*. Antimicrobial susceptibility testing was performed using the broth dilution method, with the results interpreted according to the Clinical and Laboratory Standards Institute M100 [19].

Footnotes: *K. quasipneumoniae*, *K. quasipneumoniae* subsp. *similipneumoniae*; NA, not applicable; TMP-SMX, trimethoprim/sulfamethoxazole.

ESBL genotypes of *K. pneumoniae* and *K. quasipneumoniae* subsp. *similipneumoniae*

The ESBL genotypes of *K. pneumoniae* and *K. quasipneumoniae* are detailed in Table 3. Overall, the CTX-M-1 and CTX-M-9 groups were the predominant genotypes. The CTX-M-1 group was the most prevalent in both *K. pneumoniae* (83/135, 61.5%) and *K. quasipneumoniae* (3/4, 75.0%). Among the CTX-M-1 groups, CTX-M-1 alone was only observed in *K. quasipneumoniae* (3/4, 75.0%) and not in *K. pneumoniae* ($P < 0.0001$). The coexisting CTX-M-1, SHV, and TEM were the most frequently detected genotypes in *K. pneumoniae* (76/135, 56.3%) but was not found in *K. quasipneumoniae* ($P = 0.04$).

Approximate locations for Table 3.

Table 3. ESBL genotypes of *K. pneumoniae* and *K. quasipneumoniae* subsp. *similipneumoniae*

**ESBL genotype, % (no.)**	**Percentage of cohort (no.)**		**P value**
	***K. pneumoniae* (*n* = 135)**	***K. quasipneumoniae* (*n* = 4)**	
CTX-M-1 group	83 (61.5)	3 (75.0)	1
CTX-M-1	0 (0.0)	3 (75.0)	< 0.001
CTX-M-1 + SHV	7 (5.2)	0 (0.0)	1
CTX-M-1 + SHV + TEM	76 (56.3)	0 (0.0)	0.04
CTX-M-9 group	45 (33.3)	0 (0.0)	0.3
CTX-M-9 + SHV	37 (27.4)	0 (0.0)	0.6
CTX-M-9 + SHV + TEM	8 (5.9)	0 (0.0)	1
CTX-M-2 group	3 (2.2)	0 (0.0)	1
CTX-M-2 + SHV	3 (2.2)	0 (0.0)	1
Other	4 (3.0)	1 (25.0)	
SHV	4 (3.0)	0 (0.0)	1
SHV + TEM	0 (0.0)	1 (25.0)	0.03
CTX-M-8 group	0 (0.0)	0 (0.0)	NA
CTX-M-25 group	0 (0.0)	0 (0.0)	NA
CTX-M chimera	0 (0.0)	0 (0.0)	NA

GES	0 (0.0)	0 (0.0)	NA

Description: Table 3 presents the ESBL genotypes identified in *K. pneumoniae* and *K. quasipneumoniae* isolates. The detection of ESBL genes was conducted using multiplex PCR. The CTX-M-1 and CTX-M-9 groups emerged as the predominant genotypes, with CTX-M-1 group being the most prevalent in both *K. pneumoniae* and *K. quasipneumoniae*.

Footnotes: *K. quasipneumoniae, K. quasipneumoniae* subsp. *similipneumoniae*; NA, not applicable; TMP-SMX, trimethoprim/sulfamethoxazole.

The CTX-M-9 group was exclusively present in *K. pneumoniae* (45/135, 33.3%) and not in *K. quasipneumoniae* (P = 0.3). Within the CTX-M-9 group, coexisting CTX-M-9 and SHV were the most common genotype in *K. pneumoniae* (37/135, 27.4%). Beyond the CTX-M-1 and CTX-M-9 groups, other detected genotypes included the CTX-M-2 group (2.2% in *K. pneumoniae*), SHV alone (3.0% in *K. pneumoniae*), and coexisting SHV and TEM (25.0% in *K. quasipneumoniae*). Comprehensive data on the isolates and patients are provided in Table S2.

Antimicrobial susceptibilities of CTX-M-1 and CTX-M-9 groups in *K. pneumoniae*

The antimicrobial susceptibilities of the CTX-M-1 and CTX-M-9 groups are summarized in Table 4. Amoxicillin-clavulanate, cefmetazole, and carbapenems displayed low resistance rates. Conversely, cephems (excluding cefmetazole), fluoroquinolones, and TMP-SMX demonstrated high resistance rates. In both CTX-M-1 and CTX-M-9 groups, the resistance rates to amoxicillin-clavulanate piperacillin-tazobactam were below 5% and below 20%, respectively. The antibiotics with the lowest resistance rates were cefmetazole, imipenem, meropenem, and amikacin. The resistance rates to cefmetazole of the CTX-M-1 and CTX-M-9 groups were 0.0% and 8.9%, respectively. All isolates from both groups were susceptible to imipenem, meropenem, and amikacin.

Approximate locations for Table 4.

238 Table 4. Antimicrobial resistant isolates of CTX-M-1 and CTX-M-9 group in ESBL-producing *K. pneumoniae*

**Antibiotic**	**Resistant isolates, no. (%)**		**P value**
	**CTX-M-1 group (*n* = 83)**	**CTX-M-9 group (*n* = 45)**	
β-Lactam combination			
Amoxicillin-clavulanate	2 (2.4)	2 (4.4)	0.6
Piperacillin-tazobactam	16 (19.3)	5 (11.1)	0.3
Cephem			
Ceftazidime	61 (73.5)	3 (6.7)	< 0.001
Ceftriaxone	83 (100)	45 (100)	NA
Cefepime	74 (89.2)	20 (44.4)	< 0.001
Cefmetazole	0 (0.0)	4 (8.9)	0.01
Carbapenem			
Imipenem	0 (0.0)	0 (0.0)	NA
Meropenem	0 (0.0)	0 (0.0)	NA
Aminoglycoside			
Gentamicin	26 (31.3)	21 (46.7)	0.1
Tobramycin	23 (27.7)	0 (0.0)	< 0.001
Amikacin	0 (0.0)	0 (0.0)	NA

Fluoroquinolone			
Levofloxacin	47 (56.6)	15 (33.3)	0.02
Ciprofloxacin	66 (79.5)	18 (40.0)	< 0.001
Folate pathway			
TMP-SMX	74 (89.2)	32 (71.1)	< 0.01

Description: Table 4 presents a summary of the antimicrobial susceptibility patterns of the CTX-M-1 and CTX-M-9 groups. These groups showed low resistance to amoxicillin-clavulanate, cefmetazole, and carbapenems, while showing high resistance to fluoroquinolones and TMP-SMX. Notably, the CTX-M-1 group exhibited higher resistance to levofloxacin, ciprofloxacin, and TMP-SMX compared to the CTX-M-9 group.

Footnotes: NA, not applicable; TMP-SMX, trimethoprim/sulfamethoxazole.

The resistance rate to gentamicin exceeded 31% (31.3% in the CTX-M-1 group and 46.7% in the CTX-M-9 group). The CTX-M-1 group exhibited a 27.7% resistance rate to tobramycin, while the CTX-M-9 group showed no resistance to tobramycin ($P < 0.001$). The resistance rates to levofloxacin and ciprofloxacin were 56.6% in the CTX-M-1 group and 33.3% in the CTX-M-9 group, and 79.5% in the CTX-M-1 group and 40.0% in the CTX-M-9 group, respectively. Notably, the CTX-M-1 group had higher resistance rates than the CTX-M-9 group for both levofloxacin ($P = 0.02$) and ciprofloxacin ($P < 0.001$). The resistance rate to TMP-SMX was 89.2% in the CTX-M-1 group and 71.1% in the CTX-M-9 group ($P = 0.01$).

Prevalence of AmpC-production in ESBL-producing *Klebsiella* and AmpC genotypes

The results of the screening test using cefoxitin disks showed that 16 *K. pneumoniae* (11.9% of *K. pneumoniae*) and one *K. quasipneumoniae* isolates were positive for AmpC screening (Table 1). In contrast, no isolates were found to be positive for carbapenemase screening for both *K. pneumoniae* (0.0%, 95% CI 0-2.7) and *K. quasipneumoniae* (0.0%, 95% CI 0-60.2).

Table 5 shows the percentage of AmpC production in ESBL-producing *Klebsiella* and AmpC genotypes. AmpC-genotype test results showed that only one *K. pneumoniae* isolate was positive for the AmpC genotype (0.7% of *K. pneumoniae*, 95% CI 0-4.1), and this genotype was DHA. The DHA-positive isolate harbored the ESBL genotype consisting of coexisting CTX-M-9 and SHV. The remaining 15 AmpC screening positive *K. pneumoniae* were negative for all AmpC genotypes (11.9% of *K. pneumoniae*, 95% CI 6.4-17.7). One AmpC screening positive *K. quasipneumoniae* was negative for all AmpC genotypes.

Approximate locations for Table 5.

Table 5. AmpC genotypes of screening positive *Klebsiella* isolates

**AmpC genotypes**	**No. of strains**	
	**Screening positive *K. pneumoniae* (*n* = 16)**	**Screening positive *K. quasipneumoniae* (*n* = 1)**
CIT	0	0
FOX	0	0
ACT	0	0
DHA	1	0
ACC	0	0
MOX	0	0
All negative	15	1

Description: Table 5 details the percentages of AmpC production and the associated genotypes in ESBL-producing *Klebsiella*. Screening tests using cefoxitin disks identified 16 *K. pneumoniae* (11.9%) and one *K. quasipneumoniae* subsp. *similipneumoniae* isolate as positive. Multiplex PCR revealed a single *K. pneumoniae* isolate (0.7%) carrying the DHA AmpC gene, which also harbored the CTX-M-9 and SHV.

Footnotes: *K. quasipneumoniae, K. quasipneumoniae* subsp. *similipneumoniae*.

Discussion

In our study of ESBL-producing *K. pneumoniae* clinical isolates from two regions in Japan, we identified 135 *K. pneumoniae* and 4 *K. quasipneumoniae* isolates. The predominant ESBL genotypes were the CTX-M group, and there were few instances of AmpC co-production.

K. pneumoniae is predominantly found among ESBL-producing *Klebsiella* isolates worldwide. In the United States [15] and Norway [8], the prevalence of ESBL-producing *K. pneumoniae* is reported to be between 98–99%, with ESBL-producing *K. quasipneumoniae* and *K. variicola* accounting for only 0.5–1.0%. In South and Southeast Asia, *K. pneumoniae* represents 92% of isolates, *K. quasipneumoniae* 7.6%, and *K. variicola* 0% [22]. Consistent with these reports, our study found *K. pneumoniae* to be the most predominant (97.1%), followed by *K. quasipneumoniae* (2.9%), with no *K. variicola* isolates identified. Both previous studies and our findings highlight that *K. pneumoniae* is a focal point in the antimicrobial resistance within the *Klebsiella* species. ESBL-producing *K. quasipneumoniae* is more prevalent in South and Southeast Asia than in the United States, Norway, and Japan, although the reasons for this disparity are not clear. Further research is needed to understand the distribution of ESBL-producing *K. quasipneumoniae*.

In Japan, the CTX-M1 group is the primary ESBL genotype in ESBL-producing *Klebsiella*, followed by the CTX-M9 group. Our findings align with the predominance of the CTX-M1 group in the United States [5], Canada [6], Europe [7,8], Asia [9], and Africa [10]. Notably, our cohort showed a substantial presence of the CTX-M9 group, consistent with the isolates collected in Japan during 2018 [9]. In contrast, the frequency of the CTX-M9 group in ESBL-producing *Klebsiella* was 3–4% in the USA [5] and France [7], and 11–15% in Canada [6] and Africa [10]. Although these studies were conducted in different years, precluding simple

comparisons, ongoing surveillance of genotypes in ESBL-producing *Klebsiella* is essential to monitoring the emergence of new resistant clones across various regions.

Carbapenems are effective against ESBL-producing isolates and play a vital role in treating the infections they cause [23–25]. In our cohort, ESBL-producing *Klebsiella* isolates exhibited 100% sensitivity to carbapenems. Cefmetazole has been reported to have similar clinical efficacy to carbapenems in treating invasive urinary tract infections caused by ESBL-producing *Escherichia coli* [26]. Cefmetazole may also prove useful in the treatment of invasive urinary tract infections caused by ESBL-producing *Klebsiella*. However, four isolates (3%) showed resistance to cefmetazole, and one of these had the AmpC gene. These findings highlight the need for antimicrobial susceptibility testing and consideration of AmpC production when choosing cefmetazole for treating ESBL-producing *Klebsiella*.

Amoxicillin-clavulanate, quinolones, and TMP-SMX are potential oral antimicrobial options [23–25]. The resistance rate to amoxicillin-clavulanate was 5.2% in ESBL-producing *K. pneumoniae* and 0% in ESBL-producing *K. quasipneumoniae*, indicating high susceptibility in both species. In contrast, resistance rates to levofloxacin, ciprofloxacin, and TMP-SMX were high, particularly in the CTX-M1 group, which exhibited greater resistance to these antimicrobials compared to the CTX-M9 group. These results suggest that quinolone and TMP-SMX resistance in ESBL-producing *Klebsiella* is a significant concern, particularly within the CTX-M1 group.

We identified only one instance of ESBL- and AmpC-producing *K. pneumoniae*, representing 0.7% of the isolates, which harbored the DHA gene. Thus, ESBL- and AmpC-producing *K. pneumoniae* is uncommon in Japan. In the United States [5] and Denmark [27], the prevalence of ESBL- and AmpC-producing *K. pneumoniae* was 3–4%. A notably higher prevalence was observed in Iran [11], where 19 AmpC genes were detected among 52 ESBL-producing

Klebsiella isolates. These findings highlight the regional variations in the rate of ESBL- and Amp-producing *K. pneumoniae*. Reports from Iran indicate a significant concern regarding the co-production of ESBL and AmpC and its implications for antimicrobial resistance. However, regions where *K. pneumoniae* isolates co-producing ESBL and AmpC are prevalent seem to be limited.

Our study is subject to two primary limitations concerning the study site and number of isolates. First, the study was conducted in two areas in Japan, and the prevalence of *Klebsiella* species, antimicrobial resistance, and ESBL genotypes varies across different countries and regions. Therefore, the trends observed in our study may not be generalizable to other settings. The second limitation relates to the limited number of *K. quasipneumoniae* isolates available. Owing to the small sample size of *K. quasipneumoniae*, particularly regarding antimicrobial susceptibility and ESBL genotypes, we were unable to conduct a comprehensive comparison with *K. pneumoniae*. Furthermore, we were unable to evaluate ESBL-producing *K. variicola*, which is expected to be rare in Japan. Given these limitations, future research incorporating additional areas and larger sample sizes, particularly for *K. quasipneumoniae* and *K. variicola*, is recommended.

We analyzed ESBL-producing *K. pneumoniae* and *K. quasipneumoniae* clinical isolates from Japan, focusing on their antimicrobial resistance, ESBL genotypes, and AmpC production rates. In conclusion, 97.1% of the isolates were *K. pneumoniae*, predominantly exhibiting the CTX-M1 and CTX-M9 groups. Notably, the CTX-M1 group exhibited significant resistance to quinolones and TMP-SMX. AmpC-producing isolates were rare, constituting only 0.7% of the ESBL-producing *K. pneumoniae*.

References

[1] Adeolu M, Alnajar S, Naushad S, S Gupta R. Genome-based phylogeny and taxonomy of the “Enterobacteriales”: proposal for Enterobacterales ord. nov. divided into the families Enterobacteriaceae, Erwiniaceae fam. nov., Pectobacteriaceae fam. nov., Yersiniaceae fam. nov., Hafniaceae fam. nov., Morganellaceae fam. nov., and Budviciaceae fam. nov. Int J Syst Evol Microbiol. 2016;66:5575–99. doi: 10.1099/ijsem.0.001485.

[2] Wyres KL, Lam MMC, Holt KE. Population genomics of *Klebsiella pneumoniae*. Nat Rev Microbiol. 2020;18:344–59. doi: 10.1038/s41579-019-0315-1.

[3] Wyres KL, Hawkey J, Hetland MAK, Fostervold A, Wick RR, Judd LM, et al. Emergence and rapid global dissemination of CTX-M-15-associated *Klebsiella pneumoniae* strain ST307. J Antimicrob Chemother. 2019;74:577–81. doi: 10.1093/jac/dky492.

[4] Peirano G, Chen L, Kreiswirth BN, Pitout JDD. Emerging Antimicrobial-Resistant High-Risk *Klebsiella pneumoniae* Clones ST307 and ST147. Antimicrob Agents Chemother. 2020;64: e01148–20. doi: 10.1128/AAC.01148-20.

[5] Castanheira M, Kimbrough JH, DeVries S, Mendes RE, Sader HS. Trends of β-Lactamase Occurrence Among *Escherichia coli* and *Klebsiella pneumoniae* in United States Hospitals During a 5-Year Period and Activity of Antimicrobial Agents Against Isolates Stratified by β-Lactamase Type. Open Forum Infect Dis. 2023;10:ofad038. doi: 10.1093/ofid/ofad038.

[6] Denisuik AJ, Karlowsky JA, Adam HJ, Baxter MR, Lagacé-Wiens PRS, Mulvey MR, et al. Dramatic rise in the proportion of ESBL-producing *Escherichia coli* and *Klebsiella pneumoniae* among clinical isolates identified in Canadian hospital

laboratories from 2007 to 2016. J Antimicrob Chemother. 2019;74:iv64–71. doi: 10.1093/jac/dkz289.

[7] Fils PEL, Cholley P, Gbaguidi-Haore H, Hocquet D, Sauget M, Bertrand X. ESBL-producing *Klebsiella pneumoniae* in a University hospital: Molecular features, diffusion of epidemic clones and evaluation of cross-transmission. PLoS One. 2021;16:e0247875. doi: 10.1371/journal.pone.0247875.

[8] Fostervold A, Hetland MAK, Bakksjø R, Bernhoff E, Holt KE, Samuelsen Ø, et al. A nationwide genomic study of clinical *Klebsiella pneumoniae* in Norway 2001-15: introduction and spread of ESBLs facilitated by clonal groups CG15 and CG307. J Antimicrob Chemother. 2022;77:665–74. doi: 10.1093/jac/dkab463.

[9] Kakuta N, Nakano R, Nakano A, Suzuki Y, Masui T, Horiuchi S, et al. Molecular characteristics of extended-spectrum β-lactamase-producing *Klebsiella pneumoniae* in Japan: Predominance of CTX-M-15 and emergence of hypervirulent clones. Int J Infect Dis. 2020;98:281–6. doi: 10.1016/j.ijid.2020.06.083.

[10] Breurec S, Guessennd N, Timinouni M, Le TAH, Cao V, Ngandjio A, et al. *Klebsiella pneumoniae* resistant to third-generation cephalosporins in five African and two Vietnamese major towns: multiclonal population structure with two major international clonal groups, CG15 and CG258. Clin Microbiol Infect. 2013;19:349–55. doi: 10.1111/j.1469-0691.2012.03805.x.

[11] Rizi KS, Mosavat A, Youssefi M, Jamehdar SA, Ghazvini K, Safdari H, et al. High prevalence of blaCMY AmpC beta-lactamase in ESBL co-producing *Escherichia coli* and *Klebsiella* spp. clinical isolates in the northeast of Iran. J Glob Antimicrob Resist. 2020;22:477–82. doi: 10.1016/j.jgar.2020.03.011.

[12] Brisse S, Passet V, Grimont PAD. Description of *Klebsiella quasipneumoniae* sp. nov., isolated from human infections, with two subspecies, *Klebsiella quasipneumoniae* subsp. *quasipneumoniae* subsp. nov. and *Klebsiella quasipneumoniae* subsp. *similipneumoniae* subsp. nov., and demonstration that *Klebsiella singaporensis* is a junior heterotypic synonym of *Klebsiella variicola*. Int J Syst Evol Microbiol. 2014;64:3146–52. doi: 10.1099/ijs.0.062737-0.

[13] Chew KL, Octavia S, Lai D, Lin RTP, Teo JWP. Genomic Characterization of *Klebsiella quasipneumoniae* from Clinical Specimens in Singapore. Antimicrob Agents Chemother. 2021;65:e0041221. doi: 10.1128/AAC.00412-21.

[14] Rodríguez-Medina N, Barrios-Camacho H, Duran-Bedolla J, Garza-Ramos U. *Klebsiella variicola*: an emerging pathogen in humans. Emerg Microbes Infect. 2019;8:973–88. doi: 10.1080/22221751.2019.1634981.

[15] Long SW, Linson SE, Ojeda Saavedra M, Cantu C, Davis JJ, Brettin T, et al. Whole-Genome Sequencing of Human Clinical *Klebsiella pneumoniae* Isolates Reveals Misidentification and Misunderstandings of *Klebsiella pneumoniae*, *Klebsiella variicola*, and *Klebsiella quasipneumoniae*. mSphere. 2017;2:e00290-17. doi: 10.1128/mSphereDirect.00290-17.

[16] Jarlier V, Nicolas MH, Fournier G, Philippon A. Extended broad-spectrum beta-lactamases conferring transferable resistance to newer beta-lactam agents in Enterobacteriaceae: hospital prevalence and susceptibility patterns. Rev Infect Dis. 1988;10:867–78. doi: 10.1093/clinids/10.4.867.

[17] Tzelepi E, Giakkoupi P, Sofianou D, Loukova V, Kemeroglou A, Tsakris A. Detection of Extended-Spectrum β-Lactamases in Clinical Isolates of *Enterobacter cloacae* and

Enterobacter aerogenes. J Clin Microbiol. 2000;38:542–6. doi: 10.1128/jcm.38.2.542-546.2000.

[18] Bridel S, Watts SC, Judd LM, Harshegyi T, Passet V, Rodrigues C, et al. *Klebsiella* MALDI TypeR: a web-based tool for *Klebsiella* identification based on MALDI-TOF mass spectrometry. Res Microbiol. 2021;172:103835. doi: 10.1016/j.resmic.2021.103835.

[19] Clinical and Laboratory Standards Institute. Performance standards for antimicrobial susceptibility testing. M100 33th edition. CLSI. 2023.

[20] European Committee on Antimicrobial Susceptibility Testing. The EUCAST guideline on detection of resistance mechanisms v 2.0. European Committee on Antimicrobial Susceptibility Testing. 2017. Available from: https://www.eucast.org/fileadmin/src/media/PDFs/EUCAST_files/Resistance_mechanisms/EUCAST_detection_of_resistance_mechanisms_170711.pdf.

[21] Kanda Y. Investigation of the freely available easy-to-use software “EZR” for medical statistics. Bone Marrow Transplant. 2013;48:452–8. doi: 10.1038/bmt.2012.244.

[22] Wyres KL, Nguyen TNT, Lam MMC, Judd LM, van Vinh Chau N, Dance DAB, et al. Genomic surveillance for hypervirulence and multi-drug resistance in invasive *Klebsiella pneumoniae* from South and Southeast Asia. Genome Med. 2020;12:11. doi: 10.1186/s13073-019-0706-y.

[23] Rodríguez-Baño J, Gutiérrez-Gutiérrez B, Machuca I, Pascual A. Treatment of infections caused by extended-spectrum-beta-lactamase-, AmpC-, and carbapenemase-producing Enterobacteriaceae. Clin Microbiol Rev. 2018;31:e00079–17. doi: 10.1128/CMR.00079-17.

[24] Paul M, Carrara E, Retamar P, Tängdén T, Bitterman R, Bonomo RA, et al. European Society of Clinical Microbiology and Infectious Diseases (ESCMID) guidelines for the treatment of infections caused by multidrug-resistant Gram-negative bacilli (endorsed by European society of intensive care medicine). Clin Microbiol Infect. 2022;28:521–47. doi: 10.1016/j.cmi.2021.11.025.

[25] Tamma PD, Aitken SL, Bonomo RA, Mathers AJ, van Duin D, Clancy CJ. Infectious Diseases Society of America 2022 Guidance on the Treatment of Extended-Spectrum β-lactamase Producing Enterobacterales (ESBL-E), Carbapenem-Resistant Enterobacterales (CRE), and Pseudomonas aeruginosa with Difficult-to-Treat Resistance (DTR-P. aeruginosa). Clin Infect Dis. 2022;75:187–212. doi: 10.1093/cid/ciac268.

[26] Hayakawa K, Matsumura Y, Uemura K, Tsuzuki S, Sakurai A, Tanizaki R, et al. Effectiveness of cefmetazole versus meropenem for invasive urinary tract infections caused by extended-spectrum β-lactamase-producing *Escherichia coli*. Antimicrob Agents Chemother. 2023;67:e0051023. doi: 10.1128/aac.00510-23.

[27] Hansen SK, Kaya H, Roer L, Hansen F, Skovgaard S, Justesen US, et al. Molecular characterization of Danish ESBL/AmpC-producing *Klebsiella pneumoniae* from bloodstream infections, 2018. J Glob Antimicrob Resist. 2020;22:562–7. doi: 10.1016/j.jgar.2020.05.014.

Supporting Information

S1 Table. Data on the isolates of *Klebsiella* MALDI TypeR results

S2 Table. Data on the isolates of ESBL genotypes and antimicrobial susceptibilities

Click here to access/download
Supporting Information
Supplemental material (Table S1 and S2).xlsx
