# Supplementary material for: Antimicrobial resistance and AmpC production in ESBL-producing Klebsiella pneumoniae and Klebsiella quasipneumoniae: A retrospective study in Japanese clinical isolates
Source: PLoS One. 2024 May 14;19(5):e0303353. doi: 10.1371/journal.pone.0303353 (PMC11093370; doi:10.1371/journal.pone.0303353)
Supplement: Attachment — Submitted filename: Reviewers comment_ Rahab Charles Amaza_Feb 2024.docx [file pone.0303353.s003.docx]

**Antimicrobial resistance and AmpC production in ESBL-producing *Klebsiella pneumoniae* and *K. quasipneumoniae* subsp*. similipneumoniae*: A retrospective study in Japanese clinical isolates**

**General comments**

1. This is an important study with interesting findings. It is quite commendable and will add to the body of knowledge.
2. Author should kindly address issues raised as outlined below.

**Title**

1. Scientific names of organisms (bacteria) should be italicized and spelt out in full at the first instance/mention and can be subsequently abbreviated

**Abstract**

1. The abstract does not have a flow and should follow the IMRAD structure, please note
2. Line 20- Spell out ‘’ESBL’’ in full and use as an acronym in subsequent mentions
3. Line 23- ‘’The’’ isolates were identified using….
4. Line 24-“The ESBL genes in ‘’the’’ isolates…

**Introduction**

5. Lines 83-85- ‘Maintain consistency with your numbers, you either spell out in figures or words e.g ninety ; 90 or vice-versa, 139; One hundred and thirty nine. Line 111 as well
6. For context, it is important to highlight the prevalence of ESBL-producing K. pneumoniae both globally, just as you have done, and locally in Japan, not just Asia, as you mentioned
7. Line 100- Rephrase sentence to ‘’The tool obtains the mass spectra of isolates and analyzing them…
8. Line 90- Kindly confirm, is matrix-assisted laser desorption ionization-time of flight mass spectrometry, abbreviated as “MALDI-TOF MS”? Confirm on line 99 as well
9. Line 109-118- AST; Can you include the principle/aim of the broth dilution method?
