# Supplementary material for: Antimicrobial resistance and AmpC production in ESBL-producing Klebsiella pneumoniae and Klebsiella quasipneumoniae: A retrospective study in Japanese clinical isolates
Source: PLoS One. 2024 May 14;19(5):e0303353. doi: 10.1371/journal.pone.0303353 (PMC11093370; doi:10.1371/journal.pone.0303353)
Supplement: Attachment — Submitted filename: PONE D 24 04229 Review.docx [file pone.0303353.s005.docx]

Antimicrobial resistance and AmpC production in ESBL-producing *Klebsiella pneumoniae* and *K. quasipneumoniae* subsp. *similipneumoniae*: A retrospective study in Japanese clinical isolates

**Accept after minor corrections.**

Great work!

Lines 59-62 “However, owing to the frequent misidentification of *K. quasipneumoniae* and *K. variicola* as *K. pneumoniae* in clinical settings [15], the global epidemiology of ESBL-producing *K. quasipneumoniae* and *K. variicola* is ill-understood”

Can authors get a better justification for the work, because their work was not on global level of epidemiology, in fact, no researcher can claim that. This is just a retrospective study using clinical isolates in Japan. A lot of work has been done in this area by other researchers from different countries.

Lines 63-65 “This study aims to determine the prevalence of various *Klebsiella* species, ESBL genotypes, antimicrobial resistance, and AmpC co-production in clinical isolates of ESBL-producing *K. pneumonia”*

Why not add the location? …….in Japan. Take a look at line 73 to 77 for example:

“We conducted a retrospective study on ESBL-producing *Klebsiella* in Japan. In this study, we collected clinical isolates of ESBL-producing *Klebsiella* isolates and investigated the following factors: the frequency of each *Klebsiella* species, the distribution of ESBL genotypes, the comparison of antimicrobial resistance between different *Klebsiella* species as well as between the major genotypes, and the presence of AmpC beta-lactamase.”

Lines 85-87 “At Kameda Medical Center, ninety isolates were obtained between February 2020 and November 2023, while forty-nine isolates were collected at Sapporo Clinical Laboratory from October to November 2023.”

Why the disparity in the date of isolates collection? Any scientific backing for this action?

Lines 131 – 136 Detection of AmpC and carbapenemase genotypes was based on whose protocol? Properly reference the method used for reproducibility

Lines 146 and 147 “Statistical analysis was conducted using EZR version 1.54 [21], with statistical significance set at P < 0.05.”

Now, going through the tables (1 and 2), I did not see any significance indicated by superscripts. It is obvious there were a couple of significant differences. Authors should indicate such on the tables.

Lines 160-164 “Most of the ESBL-*Klebsiella* isolates were obtained from urine specimens (74/139, representing 53.2%), followed by sputum (35/139, 25.2%), and blood (15/139, 10.8%). Using *Klebsiella* MALDI TypeR for identification, 135 isolates (97.1%, [95% CI, 92.8-99.2%]) were classified as *K. pneumoniae* and four (2.9%, [95% CI, 0.8-7.2%]) as *K. quasipneumoniae* subsp. *Similipneumoniae”*.

Authors should render this as “There were significant differences in ESBL-Klebsiella isolates obtained from …..”
