# Supplementary material for: Antimicrobial resistance and AmpC production in ESBL-producing Klebsiella pneumoniae and Klebsiella quasipneumoniae: A retrospective study in Japanese clinical isolates
Source: PLoS One. 2024 May 14;19(5):e0303353. doi: 10.1371/journal.pone.0303353 (PMC11093370; doi:10.1371/journal.pone.0303353)
Supplement: Attachment — Submitted filename: Response to Reviewers.docx [file pone.0303353.s006.docx]

**Response to the Reviewers’ Comments**

We thank the reviewers for their valuable comments. Their suggestions have been incorporated into our revised manuscript. Below, we present our responses to each comment, along with the corresponding revisions in the manuscript. The reviewers' comments are in italics, and our responses are in standard text.

**Reviewer #1**

**Title**

- Scientific names of organisms (bacteria) should be italicized and spelt out in full at the first instance/mention and can be subsequently abbreviated.

Thank you for your comment. We have revised the scientific name "*K. quasipneumoniae*" to be italicized and spelled out in full at its first mention, in accordance with scientific conventions. Subsequent mentions of the organism are abbreviated accordingly.

**Abstract**

- The abstract does not have a flow and should follow the IMRAD structure, please note.

We thank the reviewers for their valuable comments. We have reviewed the abstract and revised the text of the introduction. We have also revised the structure of the abstract with additional headings for each content to make it easier for readers to understand:

Abstract, Introduction

The study of *Klebsiella quasipneumoniae*, *Klebsiella variicola*, and AmpC production in extended-spectrum β-lactamase (ESBL)-producing *Klebsiella* in Japan is limited, and existing data are insufficient. This study aims to characterize *Klebsiella* species, determine AmpC production rates, and analyze antimicrobial resistance patterns in ESBL-producing *Klebsiella* isolates in Japan.

- Line 20- Spell out ‘’ESBL’’ in full and use as an acronym in subsequent mentions.

We have defined ESBL in the revised manuscript.

- Line 23- ‘’The’’ isolates were identified using…
- Line 24-“The ESBL genes in ‘’the’’ isolates…

We thank the reviewer for the valuable comments. We have revised the sections as suggested by the reviewer.

**Introduction**

- Lines 83-85- ‘Maintain consistency with your numbers, you either spell out in figures or words e.g ninety ; 90 or vice-versa, 139; One hundred and thirty nine. Line 111 as well.

We acknowledge the reviewer's observation regarding inconsistency in number formatting. Initially, we adopted a style where we spelled out numbers less than 10 and used numerals for numbers greater than 10, alongside specific contexts where numerals were preferred (e.g., at the beginning of sentences, numbers with units of measurement, statistical data, etc.). However, we acknowledge that this consistency was not maintained throughout the manuscript. In response to this issue, we have thoroughly reviewed the numbers and made necessary revisions to ensure consistency. Below is an example illustrating the corrective measures taken:

Lines 121-122: The test encompassed 14 antibiotics, including amoxicillin-clavulanate, piperacillin-tazobactam…

- For context, it is important to highlight the prevalence of ESBL-producing K. pneumoniae both globally, just as you have done, and locally in Japan, not just Asia, as you mentioned.

We appreciate the reviewer's suggestion and have incorporated it into the introduction section. Specifically, we have highlighted the epidemiology of ESBL-producing *K. pneumoniae* in Japan to provide context on a local level. The revised text now reads: "Among the isolates of ESBL-producing *K. pneumoniae* in Japan, CTX-M-15 is the predominant genotype" (lines 61-62). We believe this addition enhances the understanding of the significance of our study by encompassing both global and local prevalence data. We sincerely thank the reviewers for their valuable input.

- Line 100- Rephrase sentence to ‘’The tool obtains the mass spectra of isolates and analyzing them…

We have rephrased the sentence as per the comment.

- Line 90- Kindly confirm, is matrix-assisted laser desorption ionization-time of flight mass spectrometry, abbreviated as “MALDI-TOF MS”? Confirm on line 99 as well.

We appreciate the reviewer's attention to detail and clarification regarding the abbreviation "MALDI-TOF MS." Indeed, the correct phrase in the section highlighted is "MALDI-TOF MS," and we have ensured its accuracy. Additionally, we noted the infrequent usage of this abbreviation throughout the manuscript and have therefore opted to remove it where appropriate for consistency.

Regarding line 99, we have retained the phrase "*Klebsiella* MALDI Type R" as it accurately describes the context within the manuscript. We believe this maintains clarity and consistency in our terminology. We sincerely thank the reviewer for their diligence in ensuring accuracy.

- Line 109-118- AST; Can you include the principle/aim of the broth dilution method?

We appreciate the reviewer's suggestion regarding the inclusion of the principle and purpose of the broth dilution method. While this method is commonly used for clinical isolates, we initially omitted a detailed description assuming familiarity among readers. However, upon reflection, we recognize the importance of providing clarity, particularly for a broad audience.

As such, we have included a brief description of the broth dilution method in the Methods section to enhance understanding. The added text now outlines the principle and purpose of the method, ensuring comprehensive coverage. We believe this addition improves the accessibility of our methodology. We sincerely thank the reviewer for their valuable input.

Lines 119-121: In this assay, the bacterial solution was inoculated into microwells containing antibiotics. Following an incubation period, bacterial growth was assessed to ascertain the minimum inhibitory concentration (MIC) for each antibiotic.

**Reviewer #2**

- Lines 59-62 “However, owing to the frequent misidentification of K. quasipneumoniae and K. variicola as K. pneumoniae in clinical settings [15], the global epidemiology of ESBL-producing K. quasipneumoniae and K. variicola is ill-understood”
- Can authors get a better justification for the work, because their work was not on global level of epidemiology, in fact, no researcher can claim that. This is just a retrospective study using clinical isolates in Japan. A lot of work has been done in this area by other researchers from different countries.

We appreciate the reviewer's insight regarding the global research landscape on *K. quasipneumoniae* and *K. variicola*. It is evident that researchers worldwide have been investigating these organisms, and we acknowledge the significance of their work. In our original manuscript, we aimed to highlight regions, including Japan, that have received limited attention in previous research on *K. quasipneumoniae* and *K. variicola*. However, we recognize that our text may have inadvertently led to misunderstanding. To address this, we have revised the manuscript to focus specifically on the issues related to *K. quasipneumoniae* and *K. variicola* in Japan. This revision provides clarity and ensures alignment with the intended message of our study. We thank the reviewer for their valuable feedback, which has helped improve the clarity and accuracy of our manuscript. Specifically, we have revised the text as follows:

Lines 69-72: However, *K. quasipneumoniae* and *K. variicola* are frequently misclassified as *K. pneumoniae* in clinical settings [15]. Consequently, the epidemiology of ESBL-producing *K. quasipneumoniae* and *K. variicola* remains poorly understood in Japan.

- Lines 63-65 “This study aims to determine the prevalence of various Klebsiella species, ESBL genotypes, antimicrobial resistance, and AmpC co-production in clinical isolates of ESBL-producing K. pneumonia”
- Why not add the location?

We appreciate the reviewer's feedback and have taken steps to enhance the clarity of our manuscript. Specifically, we have added a description indicating that the study was conducted in Japan (lines 73-75). This addition provides crucial context for readers, ensuring they understand the geographic scope of our research.

Lines 73-75: In this study, we aimed to characterize *Klebsiella* species, ESBL genotypes, antimicrobial resistance patterns, and AmpC co-production in ESBL-producing *Klebsiella* isolates in the eastern and northern regions of Japan.

- Lines 85-87 “At Kameda Medical Center, ninety isolates were obtained between February 2020 and November 2023, while forty-nine isolates were collected at Sapporo Clinical Laboratory from October to November 2023.”
- Why the disparity in the date of isolates collection? Any scientific backing for this action?

We wanted to analyze all isolates from the same time at both facilities. However, the Sapporo Clinical Laboratory did not store any isolates before October 2023. Therefore, we performed our study on the stored isolates. Since the original manuscript did not include this information, we have added the following to the manuscript.

Line 97-98: Isolates collected before October 2023 were not preserved at the Sapporo Clinical Laboratory and were excluded from this study.

We have included a discussion of the impact associated with the content as part of the limitations section.

Line 317-318: Isolates from the Sapporo Clinical Laboratory could not be analyzed due to lack of preservation prior to October 2023. This absence of data may have skewed the antimicrobial susceptibility patterns and ESBL genotypes, potentially impacting the overall distribution observed.

- Lines 131 – 136 Detection of AmpC and carbapenemase genotypes was based on whose protocol? Properly reference the method used for reproducibility.

We appreciate the reviewer's attention to detail and have made necessary revisions to ensure accuracy in our manuscript. Firstly, we have corrected the title of the chapter to accurately reflect its content as "Detection of ESBL and AmpC genotypes," aligning with the methods employed. Furthermore, we utilized commercial kits for the detection of ESBL and AmpC genotypes, specifically the ESBL Genotype Detection Kit 2 and Cica GeneusR AmpC Genotype Detection Kit 2. These kits were utilized according to the protocols provided by the manufacturer, Kanto Chemical. We have updated our manuscript to reflect these details, providing proper reference to the method used for reproducibility.

Line 140-142: These kits are commercial products based on multiplex PCR for targeting ESBL or AmpC genes and were utilized following the manufacturer's instructions (Kanto Chemical).

- Lines 146 and 147 “Statistical analysis was conducted using EZR version 1.54 [21], with statistical significance set at P < 0.05.”
- Now, going through the tables (1 and 2), I did not see any significance indicated by superscripts. It is obvious there were a couple of significant differences. Authors should indicate such on the tables.

We thank the reviewers for their comments. We have revised the table to include superscript asterisks (*) to indicate significant differences where applicable.

- Lines 160-164 “Most of the ESBL-Klebsiella isolates were obtained from urine specimens (74/139, representing 53.2%), followed by sputum (35/139, 25.2%), and blood (15/139, 10.8%). Using Klebsiella MALDI TypeR for identification, 135 isolates (97.1%, [95% CI, 92.8-99.2%]) were classified as K. pneumoniae and four (2.9%, [95% CI, 0.8-7.2%]) as K. quasipneumoniae subsp. Similipneumoniae”.
- Authors should render this as “There were significant differences in ESBL-Klebsiella isolates obtained from …..”

We appreciate the insightful comments provided by the reviewers. Our primary goal was to present the characteristics of the target sample group, and we maintained the first sentence to focus on the isolated material of the target sample. However, upon consideration of the reviewer's suggestion, we agree that revising the percentage of bacterial species would enhance clarity and readability. Additionally, although not initially included in the manuscript, we conducted a one-sample proportion test on the proportion of *K. quasipneumoniae* subsp. *similipneumoniae*. The results revealed a significant difference between *K. pneumoniae* and *K. quasipneumoniae* subsp. *similipneumoniae* (p < 0.001). Based on the reviewer's suggestion, we have incorporated the following text:

Line 155-156: The proportion of identified species within ESBL-*Klebsiella* was analyzed using the one-sample proportion test.

Line 170-171: There were significant differences in ESBL-*Klebsiella* between *K. pneumoniae* and *K. quasipneumoniae* subsp. *similipneumoniae* (p < 0.001).

**Reviewer #3**

- Lines 20-21: “The aim of this study is to evaluate the prevalence of various species, ESBL genotypes, 21 antimicrobial resistance patterns, and AmpC co-production in ESBL-producing Klebsiella”.
- It is better for the authors to first introduce the basic concept of the work before going into the aim of the work. Also, the aim is too generic without a location attached to it.

We appreciate the valuable feedback provided by the reviewers. Upon review, we acknowledge that the original abstract lacked an explanation of the importance and background of the research topic. To address this, we have incorporated an explanation of the background of the research topic before stating the purpose of the study. Additionally, we have clarified that this study specifically focuses on Japan. The revised text is as follows:

Line 21-25: The study of *Klebsiella quasipneumoniae*, *Klebsiella variicola*, and AmpC production in extended-spectrum β-lactamase (ESBL)-producing *Klebsiella* in Japan is limited, and existing data are insufficient. This study aims to characterize *Klebsiella* species, determine AmpC production rates, and analyze antimicrobial resistance patterns in ESBL-producing *Klebsiella* isolates in Japan.

- Lins 54 to 56: “However, research on ESBL and AmpC co-producing K. pneumoniae is limited”. How is it limited? Internet search shows there are a lot of studies on this.
- Lines 61-62: “the global epidemiology of ESBL-producing K. quasipneumoniae and K. variicola is ill62 understood” What do you mean by global epidemiology? This work is Japan focussed.
- The work should be specific in its aims by attaching a location to it.

We appreciate the reviewer's feedback, which prompted us to reconsider the clarity of our original manuscript. Our intention in that section was to emphasize the limited extent of studies on the frequency of ESBL and AmpC co-producing *K. pneumoniae*, particularly in certain regions, and underscore the need for investigation in Japan. However, upon reflection, we acknowledge that the original wording may have led to misunderstanding. To address this, we have revised the text to clarify that the survey in Japan was indeed insufficient. The revised section now reads as follows:

Line 64-65: However, research on ESBL and AmpC co-producing *K. pneumoniae* is limited in Japan.

Line 66-73: ESBL production has also been observed in *K. quasipneumoniae* and *K. variicola* [13, 14]. However, *K. quasipneumoniae* and *K. variicola* are frequently misclassified as *K. pneumoniae* in clinical settings [15]. Consequently, the epidemiology of ESBL-producing *K. quasipneumoniae* and *K. variicola* remains poorly understood in Japan. In this study, we aimed to characterize *Klebsiella* species, ESBL genotypes, antimicrobial resistance patterns, and AmpC co-production in ESBL-producing *Klebsiella* isolates in the eastern and northern regions of Japan.

- Lines 73-74: In this study, we 74 collected clinical isolates of ESBL-producing Klebsiella isolates and investigated.
- Line 83-85: “In total, we collected 139 ESBL-producing Klebsiella isolates from two locations in Japan: Kameda Medical Center in the eastern part and Sapporo Clinical Laboratory in the northern part”
- What is the basis for this? Which sampling technique was used?

We appreciate the reviewer's attention to detail and the opportunity to clarify the basis and challenges associated with our study design. In this study, we conducted a retrospective observational study focusing on two specific institutions from which we were able to collect samples. This approach was due to practical constraints, including limitations in our research budget, which made it impractical to collect and analyze samples from all regions of Japan. Regarding the sampling technique, we intended to analyze all isolates from the same time period at the two institutions. However, we encountered a challenge at the Sapporo Clinical Laboratory, where isolates were not stored prior to October 2023. Consequently, we performed our study on the isolates stored from that period onward. As the original manuscript did not include this context, we have added this information to ensure transparency and completeness. Additionally, we have included relevant restrictions in the manuscript to address the limitations associated with our study design.

Line 97-98: Isolates collected before October 2023 were not preserved at the Sapporo Clinical Laboratory and were excluded from this study.

Line 317-318: Isolates from the Sapporo Clinical Laboratory could not be analyzed due to lack of preservation prior to October 2023. This absence of data may have skewed the antimicrobial susceptibility patterns and ESBL genotypes, potentially impacting the overall distribution observed.

All isolates isolated during routine testing during the relevant period were included, except for the second and subsequent isolates isolated from the same patient. However, the original manuscript lacked information to help the reader understand these details. Therefore, we have added the following text:

Line 94-96: All isolates were isolated during routine testing at each laboratory and stored at -80°C until the study commenced. Only the initial isolate was incorporated into the study when multiple ESBL-producing *K. pneumoniae* isolates were derived from the same patient.

- Authors should present results in a way that significant differences or otherwise as set out under the statistics section. The silence on significant differences that is obvious on the tables and body of results is obvious in the discussion too.

We appreciate the reviewer's feedback. In response to the suggestion, we have added superscript asterisks to the table to indicate where significant differences were found. This revision enhances the clarity of the table, allowing readers to easily identify the relevant findings.

Additionally, we noted that in the original manuscript, the text of the results section presented the main points both with and without significant differences. However, there was no mention of significant differences for the number of ESBL genes. To address this, we have included a sentence specifying significant differences. The added text now reads as follows:

Line 167-171: Using *Klebsiella* MALDI TypeR for identification, 135 isolates (97.1%, [95% CI, 92.8-99.2%]) were classified as *K. pneumoniae* and four (2.9%, [95% CI, 0.8-7.2%]) as *K. quasipneumoniae* subsp. *similipneumoniae*. The difference in ESBL-*Klebsiella* was found to be statistically significant between *K. pneumoniae* and *K. quasipneumoniae* subsp. *similipneumoniae* (p < 0.001).

In addition to the modifications mentioned earlier, we have also expanded the discussion section to include an analysis of significant differences observed in the results. In the original manuscript, our discussion primarily centered around antimicrobials, which are crucial in the treatment of ESBLs. However, we recognized the importance of addressing significant differences observed in other aspects of the study. To address this gap, we have added a discussion of some of the significant differences observed in the results. This addition provides a more comprehensive analysis of the findings, enhancing the overall discussion of our study. The revised text now includes the following:

Line 266-269: In terms of *Klebsiella* species, *K. pneumoniae* showed a significantly higher proportion of the CTX-M1 group with SHV and TEM than *K. quasipneumoniae*, while *K. quasipneumoniae* showed a significantly higher proportion of the CTX-M1 group only than *K. pneumoniae*.

Line 279-286: Regarding cephalosporins, the CTX-M-1 groups exhibited higher resistance rates to ceftazidime and cefepime compared to the CTX-M-9 groups, with a particularly significant difference observed for ceftazidime. This discrepancy could potentially be linked to the prevalence of TEM. Most TEM enzymes are ceftazidimases, which possess strong capabilities in degrading ceftazidime [26]. The proportion of isolates harboring TEM was substantially higher in the CTX-M-1 group (56.3%) compared to the CTX-M-9 group (5.9%). Thus, disparities in the prevalence of TEM-carrying isolates may have contributed to the variations in resistance rates to ceftazidime between the CTX-M-1 and CTX-M-9 groups.

This series of revisions facilitated the identification of significant differences in the tables and allowed for a fuller discussion of these issues. We thank the reviewers for their comments that led to this important revision.

**Reviewer #4**

- - “The aim of this study is to evaluate the prevalence of various species, ESBL genotypes,
- antimicrobial resistance patterns, and AmpC co-production in ESBL-producing Klebsiella” this is a bit confusing especially with the use of “prevalence” since the isolate were already identified and store as ESBL producing Klebsiella. I would suggest the use of “characterization” instead.

We agree with the reviewer's suggestion. After careful consideration, we have decided that "characterization" is more appropriate than "prevalence". Therefore, we have changed "prevalence" to "characterization" in the revised manuscript:

Line 73-75: In this study, we aimed to characterize *Klebsiella* species, ESBL genotypes, antimicrobial resistance patterns, and AmpC co-production in ESBL-producing *Klebsiella* isolates in the eastern and northern regions of Japan.

- - The study provides interesting findings on the ESBL and AmpC genotypes in Klebsiella species from two healthcare facilities in Japan. The methodology session however needs to provide further information on the isolate sourcing. Were the isolates obtained from a repository and storage information? I am curious to know if there were some of the Klebsiella species that were not confirmed even though they were stored as Klebsiella.

We appreciate your inquiry regarding the source and storage of the isolates used in our study. The ESBL *Klebsiella* isolates utilized in our research were preserved isolates from a storage facility. These preserved isolates were stored frozen at -80°C until they were used for testing. It is important to note that all preserved ESBL *Klebsiella* isolates were subjected to testing against the target. We recognize the significance of this information for the interpretation of the results, understanding the condition of the isolates, and assessing any potential selection bias. However, we regret that this information was not included in the original manuscript. To rectify this oversight, we have added the following text to provide clarity and transparency regarding the source and storage of the isolates:

Line 94-95：All isolates were isolated during routine testing at each laboratory and stored at -80°C until the study commenced.

We recognize the importance of the addition regarding the source and storage of the isolates, as it contributes crucial context to the interpretation of our results. Despite its brevity, this clarification significantly enhances the transparency and completeness of our manuscript. We sincerely thank the reviewer for bringing this matter to our attention, as it has allowed us to strengthen the methodological description in our study.

- - The authors need to provide acknowledgements for the facilities where the isolates were collected.

We appreciate the reviewer's comments, and we concur with the importance of acknowledging the contributions of the facility staff who assisted us in collecting the isolates. To address this, we have added a note to the acknowledgments section, expressing our gratitude to the facility staff for their invaluable assistance.

Line 333-334：Acknowledgments: We thank the laboratory technicians at Kameda Medical Center and Sapporo Clinical Laboratory for their invaluable assistance in collecting the isolates.

- - The tables are followed by a verbatim report of the results presented in the Table. This is highly repetitive. The authors need to use either of the two methods.
- - Line 244-251- This paragraph does not seem to add any real value but merely states what can be clearly observed from the table> the authors need to choose either tables or description
- - Table 5 does not add much value per se and should be expunged since the information has already been provided in line 258-264.

We agree with the reviewers' comments. It is important to use tables and text for different purposes in a paper and to avoid excessive duplication of information. We have tried to mention key results and important figures in the text. However, there was some duplication in several parts of the text. In particular, we found lines 244-251 to be duplicative and removed this text. We determined that the content presented in Table 5 was relatively small and only needed to be addressed in the text. Therefore, we have followed the reviewer's suggestion and removed Table 5. In addition, we have carefully reviewed the Results section and removed the following text.

Line 177-181: *K. pneumoniae* showed resistance to amoxicillin-clavulanate in 5.2% of cases (7/135) and to piperacillin-tazobactam in 18.5% (25/135) of them. In contrast, *K. quasipneumoniae* subsp. *similipneumoniae* did not exhibit resistance to these antibiotics. Neither *K. pneumoniae* nor *K. quasipneumoniae* subsp. *similipneumoniae* isolates were resistant to imipenem or meropenem.

Line 184-185: High resistance to fluoroquinolones and TMP-SMX was observed in both *K. pneumoniae* and *K. quasipneumoniae* subsp. *similipneumoniae*.

Line 204: Overall, the CTX-M-1 and CTX-M-9 groups were the predominant genotypes.

Line 220-223: Beyond the CTX-M-1 and CTX-M-9 groups, other detected genotypes included the CTX-M-2 group, SHV alone, and coexisting SHV and TEM (25.0% in *K. quasipneumoniae*).

This series of revisions has made the main text more focused, highlighting the main results and key data. We believe they clarify the argument of the paper. We thank the reviewer for the opportunity to make these important revisions.

- - Table 3: it is not clear what the column “other” represents. If it is refereeing to the genotypes listed below it, then there is no need for this and it should be expunged. I suggest that the table should be presented in a clearer way. for example. To use “CTX-M-1group and CTX-M-1 alone” to differentiate where they occur tother with other genotypes and where it occurs alone rather than just “CTX-M-1” Also, “SHV group” should be reported in addition to “SHV alone”

We thank the reviewers for their comments. We have incorporated the reviewers' suggestions and revised Table 3. We have removed the "Other" column. We have also revised the description of the table to clarify CTX-M-1 alone and SHV alone:

Table 3

| **ESBL genotype, % (no.)** | **Percentage of cohort (no.)** | | **P value** |
| --- | --- | --- | --- |
|  | ***K. pneumoniae***  **(*n* = 135)** | ***K. quasipneumoniae***  **(*n* = 4)** |  |
| CTX-M-1 group only | 0 (0.0) | 3 (75.0) | < 0.001^*^ |
| CTX-M-1 group + SHV | 7 (5.2) | 0 (0.0) | 1 |
| CTX-M-1 group + SHV + TEM | 76 (56.3) | 0 (0.0) | 0.04^*^ |
| CTX-M-9 group + SHV | 37 (27.4) | 0 (0.0) | 0.6 |
| CTX-M-9 group + SHV + TEM | 8 (5.9) | 0 (0.0) | 1 |
| CTX-M-2 group + SHV | 3 (2.2) | 0 (0.0) | 1 |
| SHV only | 4 (3.0) | 0 (0.0) | 1 |
| SHV + TEM | 0 (0.0) | 1 (25.0) | 0.03^*^ |
| CTX-M-8 group | 0 (0.0) | 0 (0.0) | NA |
| CTX-M-25 group | 0 (0.0) | 0 (0.0) | NA |
| CTX-M chimera | 0 (0.0) | 0 (0.0) | NA |
| GES | 0 (0.0) | 0 (0.0) | NA |

- - The authors indicated that Phenotypic ESBL testing will be done in the methodology but made no mention of it in the result session or even in the supplementary table. It may be worthwhile to state how it compares with the ESBL genotyping.

All isolates tested were positive for ESBL phenotyping and one to three genes were positive for ESBL genotyping. However, as noted in the reviewer's comments, the results of the ESBL phenotyping test were not included. In response to this issue, we have added the following text:

Line 172-174：All ESBL-*Klebsiella* isolates tested positive in the double-disk synergy test (139/139, 100%) and were found to harbor one to three β-lactamase genes.

- - The discussions section needs to be improved to avoid repetition of results rather provide in-depth interrogation of the study findings for instance, the issue of co-production of ESBL and Amp C was briefly mentioned as a major complication of AMR but needs more elaborate discussion of the public health implication of co-production in clinical management of Klebsiella infections.

We appreciate the reviewer's feedback and recognize the importance of elaborating on the public health implications of the co-production of ESBL and AmpC in *Klebsiella* infections. The main concern with this co-production is the degradation of cefmetazole, which is active against ESBL-producing bacteria.

While our original manuscript discussed cefmetazole in the context of ESBL-producing bacteria, we acknowledge that we did not sufficiently emphasize the connection between co-production of ESBL and AmpC. This omission may have hindered the clarity of our discussion on this issue.

To address this, we have revised the text to provide a more comprehensive discussion of the public health implications of co-production of ESBL and AmpC. The revised text now emphasizes the link between co-production and the degradation of cefmetazole, shedding light on the challenges it poses in clinical management of *Klebsiella* infections:

Line 301-309: The presence of ESBL- and AmpC-producing *Klebsiella* raises concerns regarding limited antibiotic options for treating infections caused by these pathogens. Cefmetazole may be a carbapenem-sparing option for certain non-serious infections caused by ESBL-producing isolates, particularly urinary tract infections [23, 28]. However, the presence of AmpC production confers resistance to cefmetazole in isolates. Given the rarity of ESBL- and AmpC-producing *K. pneumoniae* in Japan, cefmetazole may serve as a valuable alternative to carbapenems. Understanding the local distribution and characteristics of ESBL- and AmpC-producing *K. pneumoniae* is crucial for the development of effective antimicrobial therapy strategies.

This revision allowed our manuscript to focus on the issues of ESBL- and AmpC-producing *Klebsiella*. We thank the reviewer again for their useful comments.

- - The conclusion in its current state is a repetition of the results and lacks critical engagement with the study objectives, findings and implication.

We agree with the reviewer's comments. We have re-evaluated the results of our study. We have then summarized in our conclusions the main results for our research objectives and what can be interpreted from them. We have also critiqued the limitations of our study and decided to mention in our conclusions the issues that should be addressed in the future. The revised conclusions are as follows:

Line 324-330: Our study characterizes ESBL-producing *Klebsiella* isolates in Japan. Approximately 97% of these isolates are *K. pneumoniae*, with 62% harboring the CTX-M1 group. AmpC carriage of ESBL *Klebsiella* is less than 1%, and carbapenems and cefmetazole are expected to be effective antibiotics against ESBL *Klebsiella* in Japan. However, *K. pneumoniae* with the CTX-M1 group had a high rate of resistance to quinolones and TMP-SMX, and this multidrug-resistant pathogen is a concern in clinical practice. Given the limitations of the region and time period in this study, broader areas of research and ongoing monitoring are needed.

The reviewers' comments helped us to reconsider our research findings. This process provided us with a good opportunity to reconsider our research findings. We are grateful to the reviewer.

Minor revisions

- -The title gives prominence to K. quasipneumoniae subsp. Similipneumoniae even though there were very few isolates obtained and not much discussion around it in the discussion section

As noted in the reviewer's comments, there has been little mention of *similipneumoniae*, particularly a subspecies of *K. quasipneumoniae*. On the other hand, the terms *K. pneumoniae* complex are ambiguous and often misleading to the reader. Therefore, we have changed "*K. quasipneumoniae* subsp. *similipneumoniae*" to "*K. quasipneumoniae*" in the title.

- - Pg 5, line 72-80: Further information are needed in the methodology

We appreciate the reviewer's attention to the Methods section and the concern regarding potential duplication of details. In our manuscript, the beginning of the Methods section serves to outline the main implementation points of the study, providing the reader with an overview of the study's design and procedures. Detailed methods for each aspect of the study, including isolate collection, identification of *Klebsiella* isolates, antimicrobial susceptibility testing, and detection of AmpC and carbapenemase genotypes, are then described in subsequent sections. However, upon reflection, we recognized that the original text may have been misleading to the reader. To address this concern and avoid redundancy, we have revised the Methods section to include only the study location and the research ethics statement, while removing the additional introductory text.

- -Pg 6 line 92: “further identification of Klebsiella species, detection……” This is confusing as it has been stated that MALDI-TOF was used for identification.

We thank the reviewer for pointing this out. By "further identification" we meant "the *Klebsiella* MALDI TypeR". To avoid misunderstanding, we have changed "further identification of *Klebsiella* species" to "the *Klebsiella* MALDI TypeR".

- - Line 128: m-CIM…. Please write the full meaning of the acronym on first appearance

We forgot to spell m-CIM. We thank the reviewer for checking this. As stated in the methods, we planned to perform m-CIM on isolates that had an imipenem or meropenem MIC of 2 or greater. However, as shown in the manuscript results, we did not observe any isolates in this study that had imipenem or meropenem MICs greater than 2. Therefore, m-CIM was not performed in this study. We have determined that any mention of m-CIM in the manuscript could be misleading to the reader. Therefore, we have removed the following sentence regarding m-CIM:

Line 124-129: The screening test for carbapenemase was performed using the modified carbapenem inactivation method (m-CIM) against isolates with a MIC of ≥ 2 for either imipenem or meropenem, according to the Clinical and Laboratory Standards Institute M100 [19]. The m-CIM procedure and interpretation were adherent to the Clinical and Laboratory Standards Institute M100 [19].

- Line 207: “The co-existing CTX….” This sentence lacks clarity. please rephrase.
- -Line 219: “Within the CTX-M-9 group, coexisting CTX-M-9 and SHV were the most common genotype in K. pneumoniae (37/135, 27.4%).” This statement is unclear and needs to be rephrased for better clarity.

We have revised the text as follows：

- Line 208-210: The isolate harboring the CTX-M-1, SHV, and TEM were detected in 56.3% (76/135) of *K. pneumoniae* but was not found in *K. quasipneumoniae* (P = 0.04).

- Line 216-218: Among the *K. pneumoniae* isolates, 27.4% (37/135) harbored the CTX-M-9 group and SHV, while 5.9% (8/135) harbored the CTX-M-9 group, SHV, and TEM.

- Pg 4 line 64- Correct to “Antimicrobial resistance pattern…”
- - Pg 5 line 66: Add a full-stop after pneumonia
- - Line 196-198: kindly expunge as this information has been provided in the methods section
- -Line 213-215: same as above

We thank the reviewers for taking the time to review our manuscript in detail. We have incorporated the above suggestions and revised the relevant sections. In addition, the revised manuscript will be reviewed by an English language editor.　We thank the reviewers again for their many suggestions throughout the manuscript.

We are immensely grateful to the reviewers for their invaluable insights, which have played a crucial role in refining our article and improving its clarity and comprehensibility. Their dedicated efforts in providing detailed and constructive feedback have been instrumental in enhancing the quality of our manuscript.
